# Diabetes Prevalence and Incidence Among Medicare Beneficiaries — United States, 2001–2015

**DOI:** 10.15585/mmwr.mm6843a2

**Published:** 2019-11-01

**Authors:** Linda J. Andes, Yanfeng Li, Meera Srinivasan, Stephen R. Benoit, Edward Gregg, Deborah B. Rolka

**Affiliations:** ^1^Division of Diabetes Translation, National Center for Chronic Disease Prevention and Health Promotion, CDC; ^2^Division of Heart Disease and Stroke Prevention, National Center for Chronic Disease Prevention and Health Promotion, CDC; ^3^Imperial College London, England.

Diabetes affects approximately 12% of the U.S. adult population and approximately 25% of adults aged ≥65 years. From 2009 to 2017, there was no significant change in diabetes prevalence overall or among persons aged 65–79 years (*1*). However, these estimates were based on survey data with <5,000 older adults. Medicare administrative data sets, which contain claims for millions of older adults, afford an opportunity to explore both trends over time and heterogeneity within an older population. Previous studies have shown that claims data can be used to identify persons with diagnosed diabetes (*2*). This study estimated annual prevalence and incidence of diabetes during 2001–2015 using Medicare claims data for beneficiaries aged ≥68 years and found that prevalence plateaued after 2012 and incidence decreased after 2006. In 2015 (the most recent year estimated) prevalence was 31.6%, and incidence was 3.0%. Medicare claims can serve as an important source of data for diabetes surveillance for the older population, which can inform prevention and treatment strategies.

To estimate diabetes prevalence and incidence for the study years 2001–2015, the 100% claims data for 1999–2017 were obtained from the Centers for Medicare & Medicaid Services Chronic Conditions Warehouse (*3*) (1999–2017 data were required to identify claims for up to 2 years before and 2 years after each “index” year). These data include all claims for hospital inpatient and outpatient, physician/provider services (“carrier claims”), home health agency, and skilled nursing facility services. Diabetes-related claims were identified by any diagnosis code for primary diabetes (*International Classification of Diseases, Ninth Revision* [ICD-9] code 250.x or ICD-10 code E10 or E11). A prevalent case must have had at least 1) one inpatient claim in the index year or the preceding 2 years or 2) one outpatient diabetes claim in the index year and one inpatient or outpatient claim in the 2 years following the first claim (*2*). Incident cases were defined as prevalent cases with a 2-year period with no diabetes-related diagnosis codes at the beginning of the 5-year window.

Although all U.S. residents and lawful permanent residents aged ≥65 years are eligible for Medicare,* claims are only available for those who are enrolled in Medicare Part A (hospital insurance) and Part B (medical insurance), also known as fee-for-service. Because beneficiaries can switch between fee-for-service and Medicare Advantage privately managed plans during open enrollment every year, they were only included if they were enrolled in both Part A and Part B for all 60 months of a 5-year window centered on the index year, or if they died during the window and were enrolled until the date of death. Because beneficiaries must be fully enrolled for 60 months, and incident cases must have a 24-month period with no diabetes-related diagnosis codes at the beginning of the 5-year window, persons who turned 65 during the index year or in the 2 preceding years were not eligible to be in the study population. Therefore, each index year included only beneficiaries aged ≥68 years at the end of the index year. To focus on older adults, Medicare beneficiaries with a disability or who had end-stage renal disease were not included unless they were also aged ≥68 years.

Prevalence and incidence rates were stratified by age group (68–69, 70–74, 75–79, 80–84, and ≥85 years), sex, and race/ethnicity (mutually exclusive categories of white, black, Hispanic, Asian/Pacific Islander, and “other”). Race/ethnicity was as reported by the Social Security Administration and modified by a first- and last-name algorithm that identifies more Hispanic and Asian beneficiaries (*3*). Prevalences were computed by dividing the number of prevalent cases by the number of beneficiaries fully enrolled in the 5-year window for each index year. Incidences were calculated by dividing the number of incident cases by the sum of the number of beneficiaries without evidence of diabetes and incident cases fully enrolled in the 5-year window. Standard errors and confidence intervals were not reported because the margin of error for all estimates was <0.02%. SAS software (version 9.4; SAS Institute) was used to conduct statistical analyses. Joinpoint regression (version 4.7.0.0; National Cancer Institute) was used to assess trends over time.

The overall national prevalence of diabetes among Medicare fee-for-service beneficiaries increased from 23.3% in 2001 to a high of 32.2% in 2012, and then remained approximately level through 2015 (Table). Joinpoint regression yielded three significant trends for prevalence: from 2001 to 2008, average annual percentage change (APC) was +4%; from 2008 to 2012, APC declined (−1.4%); and from 2012 to 2015, APC decreased slightly (−0.7%) (Figure 1). The prevalence of diabetes was lower among whites than among other racial/ethnic groups and was higher among men (range = 24.7% [2001] to 34.6% [2013]) than among women (range = 22.3% [2001] to 30.3% [2012]). Prevalence among both men and women remained stable from their peak years through 2015; however, this relationship varied by racial/ethnic group. Among whites and Asians/Pacific Islanders, prevalence was higher in men, whereas among blacks and Hispanics, prevalence was higher in women.

**TABLE:** Prevalence and incidence of diabetes among Medicare fee-for-service beneficiaries, aged ≥68 years, by demographic characteristic and year — United States, 2001–2015

Characteristic	Index year*														
	2001	2002	2003	2004	2005	2006	2007	2008	2009	2010	2011	2012	2013	2014	2015
**Prevalence (%)^†^**															
**Overall**	**23.3**	**24.2**	**25.1**	**26.1**	**27.2**	**28.4**	**29.5**	**30.3**	**31.0**	**31.5**	**32.0**	**32.1**	**32.0**	**31.8**	**31.6**
**Sex**															
Women	22.3	23.2	24.0	24.9	25.9	26.9	28.0	28.8	29.4	29.8	30.2	30.3	30.2	29.9	29.6
Men	24.7	25.8	26.8	27.9	29.2	30.4	31.6	32.6	33.3	33.9	34.4	34.6	34.7	34.5	34.3
**Race/Ethnicity**															
White	21.3	22.2	23.1	24.2	25.3	26.4	27.5	28.2	28.8	29.2	29.6	29.7	29.6	29.4	29.2
Black	35.0	36.4	37.7	39.2	40.6	42.0	43.3	44.3	45.1	46.0	46.9	47.4	47.6	47.5	47.4
Hispanic	40.3	40.6	40.5	40.6	41.5	42.6	43.8	44.9	45.8	46.6	47.2	47.3	47.0	46.7	46.3
Asian/Pacific Islander	29.2	30.5	32.0	33.7	35.4	37.1	39.1	40.5	41.6	42.5	43.4	43.7	43.7	43.6	43.5
Other	30.5	31.5	32.5	33.5	34.7	35.8	37.5	38.5	39.4	40.1	40.6	41.1	41.4	41.5	41.6
**Age group (yrs)**															
68–69	22.4	23.4	24.2	25.0	26.0	26.9	28.0	28.8	29.3	29.6	29.8	29.9	29.7	29.2	28.7
70–74	23.6	24.6	25.5	26.5	27.6	28.8	29.9	30.6	31.2	31.7	32.1	32.0	31.8	31.5	31.2
75–79	24.6	25.6	26.5	27.6	28.8	30.0	31.1	32.0	32.7	33.3	33.9	34.1	34.0	33.8	33.6
80–84	23.9	24.9	25.9	27.0	28.2	29.5	30.8	31.7	32.4	33.0	33.6	33.9	33.9	33.8	33.8
≥85	20.7	21.5	22.3	23.2	24.3	25.5	26.7	27.7	28.4	29.0	29.6	30.0	30.2	30.1	30.2
**Incidence (%)^§^**															
**Overall**	**3.4**	**3.5**	**3.5**	**3.8**	**4.0**	**4.0**	**4.0**	**3.8**	**3.7**	**3.6**	**3.6**	**3.3**	**3.2**	**3.1**	**3.0**
**Sex**															
Women	3.2	3.2	3.3	3.6	3.7	3.8	3.8	3.6	3.5	3.4	3.3	3.1	2.9	2.8	2.8
Men	3.7	3.8	3.8	4.1	4.3	4.4	4.4	4.2	4.1	4.0	4.0	3.7	3.6	3.5	3.5
**Race/Ethnicity**															
White	3.1	3.2	3.2	3.5	3.7	3.8	3.7	3.5	3.4	3.3	3.3	3.0	2.9	2.8	2.8
Black	4.9	5.3	5.4	5.8	6.0	6.1	6.1	5.9	5.7	5.8	5.9	5.5	5.4	5.2	5.1
Hispanic	6.5	6.6	6.5	6.6	6.8	6.9	6.9	6.6	6.4	6.4	6.4	6.0	5.7	5.4	5.2
Asian/Pacific Islander	4.6	4.8	5.0	5.5	5.8	6.1	6.2	6.0	5.8	5.7	5.6	5.3	5.0	4.8	4.7
Other	4.2	4.1	4.2	4.4	4.6	4.6	4.8	4.7	4.6	4.7	4.4	4.3	4.0	3.9	3.9
**Age group (yrs)**															
68–69	3.3	3.5	3.5	3.7	3.9	4.0	4.0	3.7	3.7	3.6	3.5	3.2	3.1	3.0	3.0
70–74	3.4	3.5	3.5	3.8	4.0	4.1	4.1	3.8	3.8	3.7	3.6	3.3	3.1	3.0	3.0
75–79	3.5	3.6	3.6	3.9	4.1	4.2	4.2	3.9	3.8	3.7	3.7	3.4	3.2	3.1	3.1
80–84	3.4	3.5	3.6	3.8	4.1	4.1	4.1	3.9	3.8	3.7	3.7	3.4	3.3	3.2	3.2
≥85	3.1	3.2	3.2	3.5	3.6	3.7	3.8	3.6	3.5	3.4	3.5	3.3	3.1	3.0	3.0
**Total no., in millions**	**25.2**	**25.5**	**26.0**	**25.9**	**25.6**	**25.0**	**24.8**	**24.6**	**24.6**	**24.7**	**24.6**	**24.5**	**24.4**	**24.6**	**24.5**

* Index year indicates the year for which prevalence and incidence is reported. It is the year at the center of a 5-year data window.

^†^ Calculated by dividing the number of prevalent cases by the number of beneficiaries fully enrolled during the 5-year window for each index year.

^§^ Calculated by dividing the number of incident cases by the sum of the number of beneficiaries without evidence of diabetes and incident cases fully enrolled during the 5-year window.

**FIGURE 1:**
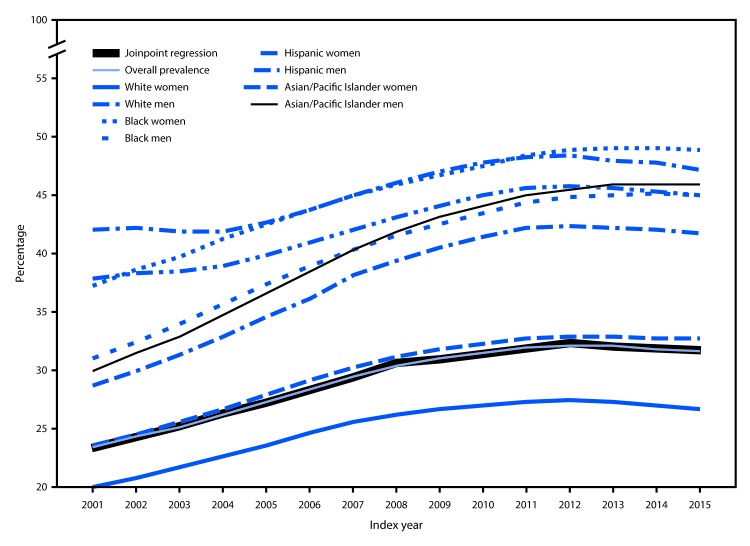
Prevalence of diabetes among Medicare fee-for-service beneficiaries aged ≥68 years — United States, 2001–2015

Two significant trends in incidence were observed. From 2001 to 2006, APC was +4.5%; after 2006, incidence decreased (APC = −3.3%) (Figure 2). Although incidence varied little by age, there were substantial differences by race/ethnicity and sex (Table). As with prevalence, incidence among whites and Asians/Pacific Islanders was higher among men, although among blacks and Hispanics, incidence was similar among men and women.

**FIGURE 2:**
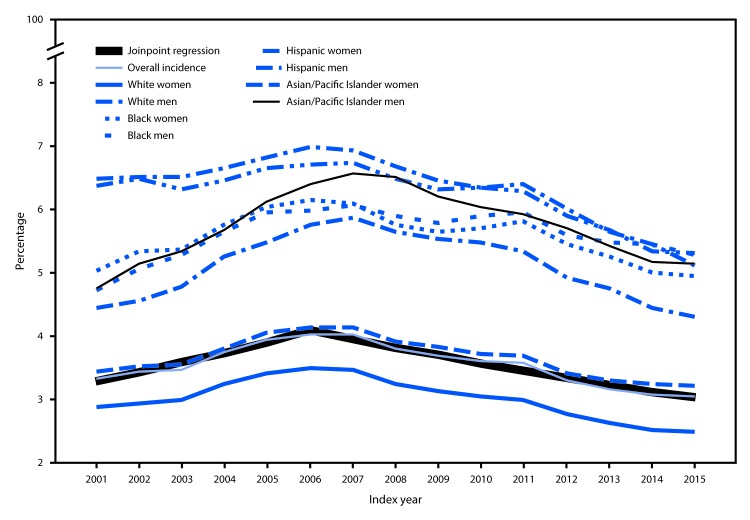
Incidence of diabetes among Medicare fee-for-service beneficiaries aged ≥68 years — United States, 2001–2015

## Discussion

This study found that, among Medicare beneficiaries, the overall prevalence of diabetes increased from 2001 to 2012 and then remained approximately stable through 2015, and that the overall incidence decreased from 2006 to 2015. During 2015, the overall prevalence and incidence of diabetes among Medicare fee-for-service beneficiaries aged ≥68 years were 31.6% and 3.0%, respectively.

These findings are consistent with survey-based estimates showing a flattening of prevalence after the year 2008 for all age groups and a decrease in incidence from 2009 to 2017 among all age groups (*4*). Several factors could explain these trends in diabetes prevalence and incidence. National data have suggested that some important risk factors, including total dietary intake, added sugar and sugar-sweetened beverage intake, and physical inactivity, might have decreased in the past decade (*5*,*6*). 

According to national survey data (*4*), the prevalence of self-reported diabetes diagnosed by a health care provider among Americans aged ≥65 years in 2017 was 20.8%, and the incidence was 0.9%. In comparison, claims-based prevalence and incidence reported in this study were substantially higher. However, both claims-based and survey-based estimates are subject to several sources of bias that might explain the difference in estimates.

The study sample might not be representative of the population of Medicare beneficiaries because claims for those enrolled in Medicare Advantage were not included in the data. However, a recent study of data from the National Health and Nutrition Examination Survey linked to Medicare enrollment data found no difference in diagnosed diabetes between fee-for-service and Medicare Advantage enrollees (*7*). In addition, the study sample might not be representative of the Medicare fee-for-service population because beneficiaries were required to be enrolled in both Part A and Part B for 60 months continuously or, if less than 60 months, then enrolled up until date of death. Also, one study recommends a clean period of ≥3 years for incidence estimations (*2*); thus, the requirement for only a 2-year period without diabetes-related diagnosis codes might have overestimated incidence. Further, diagnoses of diabetes in administrative claims data might be affected by patterns of health care utilization which are known to vary by sex and age (*8*).

Survey-based estimates might also be biased. Previous research has shown that measuring diabetes status from claims data yields higher prevalence rates than do self-reports among the same beneficiaries (*9*). Researchers compared diabetes identification from self-report on the National Health Interview Survey to that from respondents’ linked Medicare claims and found that 93.1% of beneficiaries who self-reported diabetes were also identified through their claims. In contrast, only 67.0% of beneficiaries who were identified as having diabetes through their claims also self-reported having diabetes (*9*). This suggests there might be a substantial underestimation in survey-based estimates, possibly because of respondents’ misunderstanding of survey questions or health care providers’ communication, social desirability, or from simple failures of recall in self-reports. Surveys might underestimate diabetes prevalence because they are subject to selection bias in which very sick persons do not respond. Further, surveys usually sample from noninstitutionalized adults, excluding persons who are in hospitals or nursing homes from the sampling frame while they are included in claims data. Therefore, estimated rates from surveys are expected to be lower than those for the study population.

National surveys are important for understanding the burden of diabetes (*4*), but Medicare claims provide more detailed data on the older population, who experience a higher disease burden from diabetes. These data are an important source for future diabetes surveillance in the older population to monitor disease burden over time and assess disease prevention and management activities.

**Summary**

**What is already known about this topic?**

Survey data have been crucial for diabetes surveillance, but administrative claims data from Medicare can also be used to track prevalence and incidence.

**What is added by this report?**

The prevalence of diabetes among adults aged ≥68 years has plateaued in recent years, and survey data and Medicare claims indicate that incidence has also declined. However, both prevalence and incidence obtained from Medicare fee-for-service claims are higher than those from survey data.

**What are the implications for public health practice?**

Diabetes prevalence and incidence might be higher among Medicare fee-for-service beneficiaries than that indicated by existing surveillance, which can improve efforts to monitor disease burden over time and assess disease prevention and management activities.

## Footnotes

* https://www.cms.gov/Medicare/Eligibility-and-Enrollment/OrigMedicarePartABEligEnrol/index.html.
